# Polyamine Metabolites as Biomarkers in Head and Neck Cancer Biofluids

**DOI:** 10.3390/diagnostics12040797

**Published:** 2022-03-24

**Authors:** Brian C. DeFelice, Oliver Fiehn, Peter Belafsky, Constanze Ditterich, Michael Moore, Marianne Abouyared, Angela M. Beliveau, D. Gregory Farwell, Arnaud F. Bewley, Shannon M. Clayton, Joehleen A. Archard, Jordan Pavlic, Shyam Rao, Maggie Kuhn, Peter Deng, Julian Halmai, Kyle D. Fink, Andrew C. Birkeland, Johnathon D. Anderson

**Affiliations:** 1 West Coast Metabolomics Center, University of California, Davis, CA 95616, USA; bcdefelice@ucdavis.edu (B.C.D.); ofiehn@ucdavis.edu (O.F.); 2 Department of Otolaryngology-Head and Neck Surgery, University of California, Davis, CA 95616, USA; pcbelafsky@ucdavis.edu (P.B.); cbergt@ucdavis.edu (C.D.); mgmoore@gmail.com (M.M.); mabouyared@ucdavis.edu (M.A.); ambeliveau@ucdavis.edu (A.M.B.); greg.farwell@pennmedicine.upenn.edu (D.G.F.); abewley@ucdavis.edu (A.F.B.); smclatyon@ucdavis.edu (S.M.C.); jaarchard@ucdavis.edu (J.A.A.); jodranpavlic@csus.edu (J.P.); makuhn@ucdavis.edu (M.K.); 3 Department of Radiation Oncology, University of California, Davis, CA 95616, USA; sdrao@ucdavis.edu; 4 Department of Neurology, University of California, Davis, CA 95616, USA; pbdeng@ucdavis.edu (P.D.); jhalmai@ucdavis.edu (J.H.); kdfink@ucdavis.edu (K.D.F.)

**Keywords:** head and neck cancer, biomarkers, metabolomics, polyamines, saliva, urine

## Abstract

*Background*: Novel, non-invasive diagnostic biomarkers that facilitate early intervention in head and neck cancer are urgently needed. Polyamine metabolites have been observed to be elevated in numerous cancer types and correlated with poor prognosis. The aim of this study was to assess the concentration of polyamines in the saliva and urine from head and neck cancer (HNC) patients, compared to healthy controls. *Methods:* Targeted metabolomic analysis was performed on saliva and urine from 39 HNC patient samples and compared to 89 healthy controls using a quantitative, targeted liquid chromatography mass spectrometry approach. *Results:* The metabolites N^1^-acetylspermine (ASP), N^8^-acetylspermidine (ASD) and N^1^,N^12^-diacetylspermine (DAS) were detected at significantly different concentrations in the urine of HNC patients as compared to healthy controls. Only ASP was detected at elevated levels in HNC saliva as compared to healthy controls. *Conclusion:* These data suggest that assessment of polyamine-based metabolite biomarkers within the saliva and urine warrants further investigation as a potential diagnostic in HNC patients.

## 1. Introduction

Head and neck cancer (HNC) is the seventh most common cause of cancer-related death, and accounts for approximately 6% of all cancer cases globally [1]. There are over 800,000 new cases and over 400,000 deaths from HNC annually [1,2].

Effective treatment of HNC patients is facilitated by early detection, and appropriate therapeutic interventions for each stage of cancer [3]. Unfortunately and commonly, HNC is not clinically detected until a patient presents with symptoms associated with later stage primary disease or when lymphatic metastases are palpable [4]. Such symptoms include pain, bleeding, ulceration, otalgia, and dysphagia [5]. Some reports suggest that only 30% of HNC cases are diagnosed at an early stage, while approximately 66% of patients present with advanced stage III or IV tumors at the time of diagnosis [6].

Treatment of cancer is generally most effective when the tumor burden is lowest at the primary site and when the lymphatic spread is minimal or absent [7]. Effective treatment of HNC depends on early diagnosis and surgical/chemoradiation intervention [8]. Despite the apparent advantage of early diagnosis of neoplasia, no strategy has yet proven to be a consistently effective means of diagnosing early stage HNC, and no conventional screening methods have led to decreases in mortality [9]. Recently several groups have reported promising preliminary analyses of biomarkers for the early detection of HNC. Further investigation is required to validate these studies and determine the clinical feasibility and diagnostic accuracy of the identified biomarkers [10].

The metabolome is the complete set of metabolites within a cell, tissue, or biological sample at any given time point. The metabolome can provide important insight into the pathophysiologic mechanisms of numerous diseases [11,12]. Evaluation of endogenous metabolites such as nucleic acids, amino acids, peptides, organic acids, thiols, and carbohydrates represents a valuable tool for the identification of biomarkers for multiple diseases, providing prognostic and disease monitoring insights [13]. Polyamines, which include spermidine, spermine, and putrescine, are polycationic alkylamines that are present in mammalian cells at millimolar concentrations [14]. These molecules are involved in many critical processes such as cell proliferation, nucleic acid synthesis, and cyto-protection from oxidative stress [15]. A growing body of work has established that polyamine metabolism is frequently dysregulated in cancer [16]. Several published reports have determined that elevated polyamine levels are essential for tumor progression [17].

The concentration of polyamines has been observed to be increased in the blood and urine of patients with several types of cancer including breast, colon, and prostate [18]. Elevated levels of polyamines in these biological fluids have been demonstrated to correlate with poor prognosis [19]. The polyamine levels may be attributable to the increased synthesis of these by highly proliferative cancer cells [20]. It has also been observed that elevated polyamine levels in cancer patients are often attenuated following surgical or chemoradiation eradication of tumors [21].

As polyamines are indispensable for cellular growth, the capacity of cancer tissue to produce abundant polyamines may contribute to the aggressive behavior of cancer cells and the association of poorer prognosis in patients with enhanced polyamine levels [22]. However, the presence of elevated polyamine levels in HNC patients is currently uncertain. Therefore, we decided to investigate whether specific, well-established polyamines (N1-acetylspermine (ASP), N8-acetylspermidine (ASD), and N^1^,N^12^-diacetylspermine (DAS)) are elevated in the biofluids of HNC patients. An additional advantage of such an approach is that targeted mass-spectrometry methods are more sensitive than untargeted approaches, allowing for more robust detection of metabolites.

Although they have yet to be widely utilized in routine clinical care, numerous urine-based biomarkers have been developed as alternatives or adjuncts to standard tests for the initial diagnosis of several types of cancer and assessment of recurrent disease [23]. The essential advantages of urine as a biofluid source for biomarker research and development are that its acquisition is noninvasive, and the fluid contains proteins and metabolites associated with pathophysiology [24]. In addition, urine samples can be analyzed cost effectively, and are easily stored, stable, and sterile [25]. To date, no less than six urinary biomarkers have been approved by the Federal Drug Administration for the detection and surveillance of cancer [26].

Saliva is a biological fluid comprised of approximately 99% water and 1% proteins, electrolytes and other low-molecular weight components such as metabolites [27]. Oral secretions are an acidic fluid derived from salivary glands, cellular debris, crevicular fluid, nasal/bronchial secretions, bacteria and exogenous ingested substances [28]. Saliva is largely generated from three pairs of major salivary glands (submandibular, parotid, and sublingual) as well as from 300–400 minor glands present in the oral cavity [29]. Saliva can be obtained in a facile, noninvasive, and inexpensive manner, and can reflect a patient’s physiological state. Saliva testing ostensibly allows for patients to gather their own samples at home, saving healthcare costs, and enabling a convenient way to garner multiple sequential samples. Currently, oral fluid tests have been developed for the detection of specific infectious agents (e.g., HIV, HSV, HPV, SARS-CoV-2, etc.) to evaluate metabolizer status for numerous drugs and for the detection of illicit drugs. Mucosal HNCs may also contribute cellular components to saliva, which may allow the fluid to be utilized to detect potential prognostic and disease monitoring biomarkers.

Here we establish that both p16 positive and p16 negative HNC patients have differential levels of one or more polyamines present in both saliva and urine as compared to healthy controls. These data suggest that polyamine concentrations from biofluids may serve as a diagnostic biomarker for HNC, indicating that further investigation to validate such approaches may be warranted.

## 2. Materials and Methods

### 2.1. Reagents

N^1^-acetylspermine (ASP) and N^8^-acetylspermidine (ASD) standards were purchased from Sigma-Aldrich (St. Louis, MO, USA). (N^1^,N^12^-diacetylspermine) DAS was synthesized in-house, and confirmed via liquid chromatography retention time matching and tandem MS/MS spectral-matching. Deuterium-labeled DAS (DAS-d6) was purchased from Santa Cruz Biotechnology Inc. (Dallas, TX, USA). Ammonium formate, LC-MS grade acetonitrile, water, formic acid, and methanol were purchased from Fisher Scientific (Hampton, NH, USA). Chromatographic separation was attained using an ACQUITY UPLC HSS PFP column (1.8 μm particle size, 2.1 mm, 100 mm) purchased from Waters Corporation (Milford, MA, USA). One milliliter, 96-well plates were purchased from Eppendorf (Hamburg, Germany). 96-well 0.2-micron PVDF filter plates were obtained from Agilent (Santa Clara, CA, USA). 12-[[(cyclo-hexylamino) carbonyl]amino]-dodecanoic acid (CUDA) was purchased from Cayman Chemicals (Ann Arbor, MI, USA). Urine and saliva were provided by Dr. Peter Belafsky with appropriate IRB approval (#708419).

### 2.2. Biofluid Samples

Saliva and urine samples were collected via an approved IRB protocol at the University of California Davis Medical Center in Sacramento, CA. Partners and other live-in relatives of HNC patients consented to donate the healthy saliva and urine control samples. Polyamines were evaluated in urine from 39 HNC patients and 89 healthy controls. Thirty five HNC patients and 72 healthy control saliva samples were used to evaluate polyamines concentrations. Patient and tumor data were collected from HNC patients.

### 2.3. Sample Processing

Metabolite extractions and LC-MS/MS analysis were carried out in a high-throughput manner as published previously [30], with some modification. Briefly, saliva samples were first centrifuged at 3000× *g* for 10 min to precipitate denatured mucins as described elsewhere, and 100 µL of supernatant was taken from each saliva sample. Urine sample volumes were normalized to creatinine levels prior to extraction, volumes ranging from 1 to 66 µL, and water was used to bring the final volume to 100 uL. All further steps were applied to both saliva and urine equally. An extraction solvent consisting of 1:1 mixture of aceto-nitrile:methanol and spiked with 200 pg/mL of DAS-d6 internal standard was cooled to −20^C^ and added to each sample well. Plates were capped with silicon 96-well plugs, followed by vortexing for 5 min at speed 6 of a VX-2500 vortexer (VWR, Radnor, PA, USA). Precipitated proteins were pelleted by 5 min centrifugation. Supernatants were moved to a new 96-well plate and subsequently evaporated in a EZ-2 plus centrivap (GeneVac, Ipswich, UK). One hundred microliters of a 9:1 water:acetonitrile solution spiked with 50 ng/mL of CUDA was used to resuspend samples. CUDA peak intensities were monitored throughout the analysis to ensure consistent injection volumes. Sample plates were plugged, and vortexed for 5 min, then samples were passed through a 0.2-micron PVDF filter to remove residual particulate matter. Sample plates were sealed with aluminum foil by an ALPS 3000 Microplate Sealer (Thermo Scientific, Waltham, MA, USA), stored at 4^C^ and analyzed within 48 h.

### 2.4. LC-MS/MS Analysis

**Figure 1:**
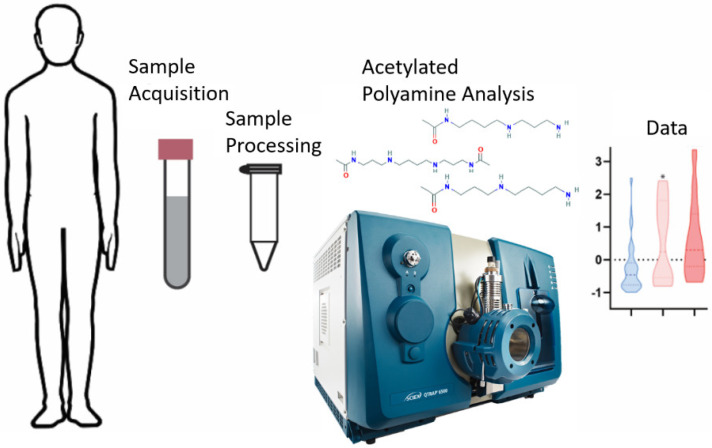
Workflow schematic of targeted metabolomics assessment of polyamine levels in urine and saliva in head and neck cancer patients as compared to healthy controls, * *p* < 0.05.

Quantification of APs was performed using a Sciex 6500+ QTRAP (Redwood City, CA, USA) mass spectrometer with electrospray ionization source. Separation was achieved using a Waters ACQUITY high strength silica (HSS) penta-fluorophenyl (PFP) ultra-performance liquid chromatography (UPLC) column (1.8 μm, 2.1 mm × 100 mm) mounted in a Waters ACQUITY I-class UPLC system (Milford, MA, USA). Mobile phase composition and gradient information have been described elsewhere [30]. Mass spectrometer ion-source and collision cell settings were optimized previously [30] for acetylated polyamines The mass spectrometer was operated in multiple reaction monitoring mode, and two ion transitions per metabolite target were monitored (Figure 1).

### 2.5. Data Processing

Data was processed using Sciex software, MultiQuant v3.0.2. A twelve-point calibration curve was created using a 3:1 serial dilution in order to quantify the target metabolites. CUDA peak areas were visually assessed to ensure complete injections of the desired volumes. The ratio of the analyte’s peak area to DAS-d6 internal standard’s peak area was utilized to generate a calibration curve for each metabolite. Calibration curves were all generated linearly, with the X variable weighted as 1/X. Acetylated polyamine concentrations were calculated via the calibration curves.

### 2.6. Statistics

Statistical analyses were calculated using SPSS v25, and graphs generated using GraphPAD Prism v8.2.1. For dual variant analyses, a Mann-Whitney U test was utilized. For multivariate analyses a Kruskal–Wallis Test was used with a Dunn’s post hoc to determine significance of intergroup differences.

## 3. Results

### 3.1. HNC Population Characteristics

A total of 39 HNC patients were included in the study, with an age range of 39–83, with a mean age of 66 years old (Table 1). We had an even distribution of early (I,II) and late (III,IV) stage HNCs (19 early stage and 20 late stage. Nine patients were p16 positive. The majority of HNCs were from the oral cavity (54%) or oropharynx (41%). The ratio of female participants to male was 16:23, respectively (Table S1). Controls samples were collected from 89 healthy individuals (59% female, 41% male), with an age range of 18–85, and with a mean age of 52 years old (Table S2).

### 3.2. Polyamine Levels in HNC-Derived Saliva

**Figure 2:**
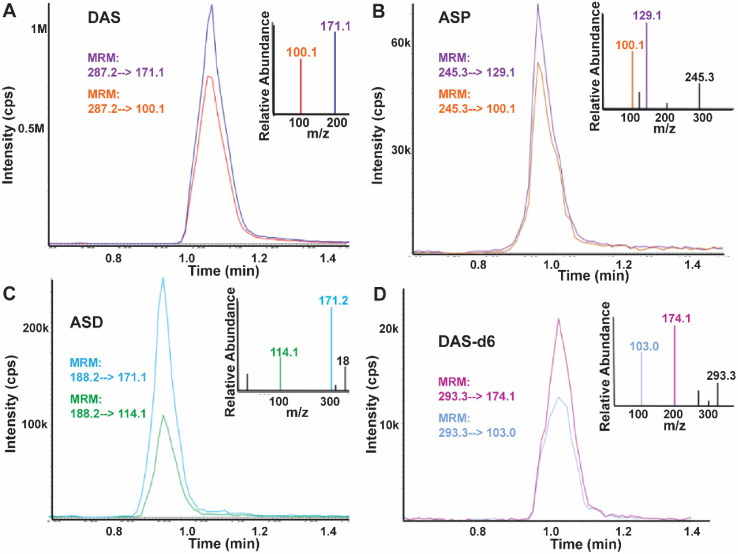
The MS^2^ spectra from metabolite standard and the generated MRM chromatograms for the three target metabolites and the isotopically labeled internal standard, chromatographic data taken from a HNC saliva sample. (**A**) N^1^,N^12^-diacetylspermine (DAS) (**B**) N^1^-acetylspermine (ASP) (**C**) N^8^-acetylspermidine (ASD) (**D**) Deuterium labeled N^1^,N^12^-diacetylspermine (DAS-d6).

To assess whether polyamines are present at elevated levels in the biofluids of HNC patients compared to healthy controls, we used a targeted metabolomics approach [30] (Figure 1). Using a novel mass spectrometry approach we quantified concentrations of N^1^-acetylspermine (ASP), N^8^-acetylspermidine (ASD), and N^1^,N^12^-Diacetylspermine (DAS) in saliva samples, as previously reported [30]. Standard curves were generated using MS^2^ spectra from each metabolite standard. Mass spectrometry spectra were also used to generate the MRM chromatograms for the three target metabolites and a stable isotope labeled internal standard (Figure 2).

**Figure 3:**
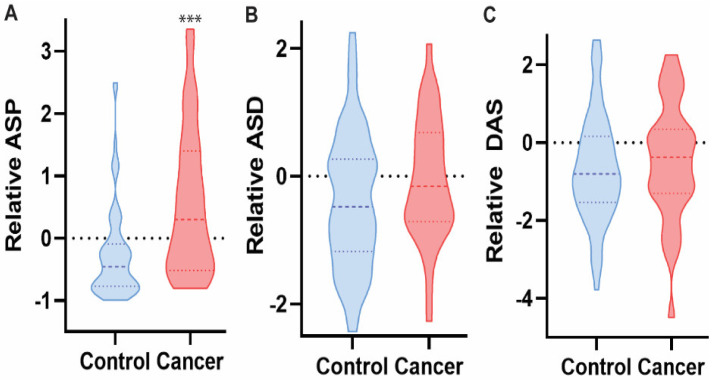
ASP, but not ASD or DAS, was significantly elevated in the saliva of HNC patients as compared to healthy controls based on targeted mass spectrometry analysis. Mann-Whitney U test, *** *p* < 0.0001, *n* = 107.

**Figure 4:**
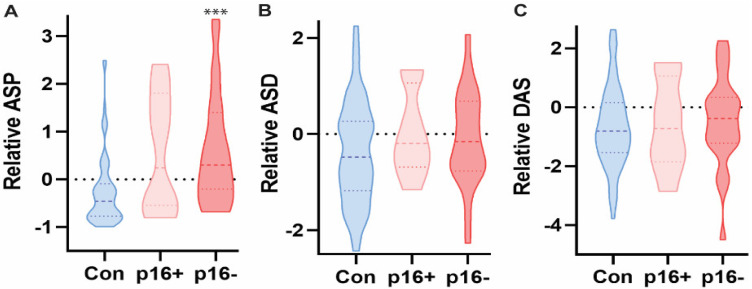
ASP, but not ASD or DAS, was significantly elevated in the saliva of p16+ populations in HNC patients as compared to healthy controls based on targeted mass spectrometry analysis. Kruskal-Wallis test with multiple comparisons across groups, *** *p* < 0.0001, *n* = 107.

A total of 107 samples were analyzed: controls *n* = 72, HNC = 35 (p16 positive *n* = 8, and p16 negative *n* = 27). Comparisons of HNC saliva to healthy control samples determined ASP to be differentially represented (*p* < 0.007) (Figure 3). ASD and DAS were not significantly different in HNC than in control saliva samples. Further analysis established a significant differential level of ASP in p16 negative HNC saliva as compared to healthy controls (*p* < 0.05, and *p* < 0.0001, respectively) (Figure 4). ASD and DAS metabolites were not enriched in HNC saliva samples as compared to controls, regardless of p16 status (Figure 4). These data suggest a small but significant enrichment of ASP in HNC derived saliva samples.

### 3.3. Polyamine Levels in HNC-Derived Urine

**Figure 5:**
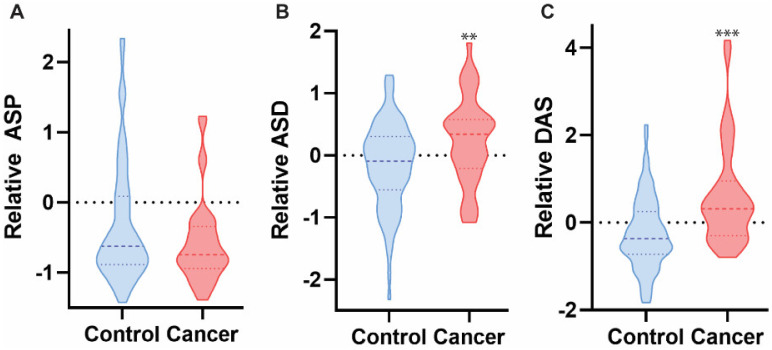
ASD and DAS were detected at significantly elevated concentrations in the urine of HNC patients as compared to healthy controls using targeted mass spectrometry approach, whereas ASP was not observed in significantly different quantities. Mann-Whitney U test, ** *p* < 0.005, *** *p* < 0.0001, *n* = 107.

**Figure 6:**
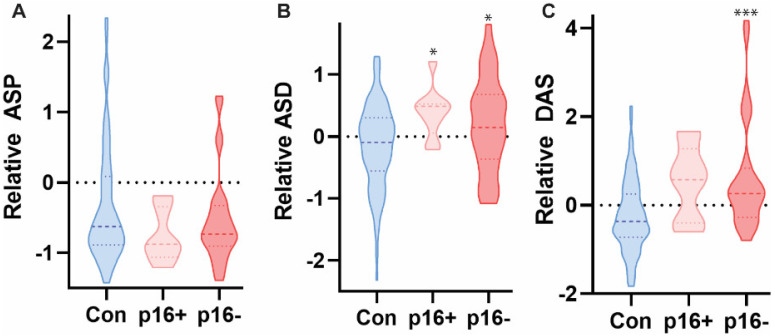
ASD and DAS, but not ASP, were significantly elevated in the urine of p16− patients, as compared to p16+ and control groups. Only ASD was significantly elevated in p16+ patients as compared across groups. Kruskal-Wallis test with multiple comparisons across groups * *p* < 0.05, *** *p* < 0.0001, *n* = 107.

Next, we used the same methodology to evaluate polyamine concentrations in urine sample isolated from both HNC patients and healthy controls. A total of 124 samples were analyzed: controls *n* = 89, HNC = 39 (p16 positive *n* = 9, and p16 negative *n* = 30). HNC urine samples contained significantly different concentrations of all three metabolites (ASP, ASD, and DAS), as compared to healthy controls (*p* < 0.03, *p* < 0.007, and *p* < 0.02, respectively) (Figure 5). Further analysis established that both ASD and DAS metabolites were enriched in p16− urine samples, as compared to healthy controls (*p* < 0.04, and *p* < 0.0001, respectively) (Figure 6). These data indicate a small but significant enrichment of the polyamines ASP, ASD, and DAS in HNC urine samples, as compared to healthy controls.

## 4. Discussion

In this study, we analyzed specific polyamines (ASP, ASD, and DAS) levels in urine and saliva biospecimens in HNC patients in comparison to normal controls. We identify elevated levels of ASP in HNC patient saliva, and elevated levels of ASD and DAS in HNC patient urine, in comparison to control patients. To our knowledge, this is the first study identifying elevated levels of ASP, ASD and DAS polyamines in saliva and urine samples of HNC patients. In sum, these initial analyses suggest a potential role for assessing polyamines as a liquid biomarkers in HNCs in further investigations.

Numerous investigations into liquid biomarkers in HNC are actively being performed; however, no technique has consistently identified usable biomarkers in HNCs. Studies assessing viral signatures in virally-associated HNCs (human papillomavirus and Epstein-Barr virus) have been successful [31,32]; unfortunately, these only represent a subset of HNCs and a majority of HNCs are not virally-associated. Additionally, many biomarkers predominantly use blood for analysis. The development of a liquid biomarker utilizing non-invasive techniques (i.e., urine or saliva) and able to identify all subsets of HNC (virally and non-virally mediated) would be of enormous value.

Elevated polyamine levels have been identified in other cancer types, including breast, colon and prostate cancers [18] and have been associated with worse prognosis in other cancers [19]. Thus, there is notable biological rationale to assess polyamines as potential biomarkers both in cancer screening and in assessing response to treatment. Published reports have indicated that polyamines and their metabolite derivatives may be useful as markers of tumor progression in lung and liver cancers [33,34]. Polyamines evaluated in urine and blood serum have also demonstrated potential as biomarkers for colon, prostate and pancreatic cancers [35,36,37,38].

Notably, initial studies analyzing polyamine levels were limited by the low sensitivity of the methodologies used. Recently developed, highly sensitive metabolomic techniques can provide more robust measurements. Thus, there is interest and a rationale for further metabolomic investigations for optimization and analysis of polyamines in HNC and other cancers. Given the noninvasive nature of saliva and urine collection, and the potential cost-effectiveness and large-scale analysis opportunities with certain metabolomic approaches, there are opportunities for developing future screening opportunities for at-risk patient populations.

There are limitations to our study. Notably, our HNC cohort was of modest size. Additional specimen collection and analysis would bolster our initial results. Nevertheless, we were able to detect differences in polyamine levels in this initial HNC cohort.

To date, the diagnostic value of polyamines in HNC have been unclear. The data in this investigation suggests that the polyamines ASP, ASD and DAS are present at significantly different concentrations in the saliva and urine of HNC patients as compared to healthy controls. These proof of concept data indicate that alterations in the presentation of the metabolites ASP, ASD and DAS in biofluids is significant, although the differentials were modest in this moderately sized study. Further larger-scale investigations would be highly valuable to provide additional support and rationale for the preclinical and clinical development of these biomarkers for screening and surveillance for HNCs.

## Figures and Tables

**Table 1:** HNC patient and tumor characteristics.

HNC Cohort	Characteristic	Number (Percentage)
	Age	66 (range: 39–83)
Gender	Male	23 (59)
	Female	16 (41)
Site	Oral Cavity	21 (54)
	Oropharynx	16 (41)
	Larynx	1 (3)
	Nasopharynx	1 (3)
Stage	I	11(28)
	II	8 (21)
	III	5 (13)
	IV	15 (38)
P16 Status	Positive	9 (23)
	Negative	30 (77)

## Data Availability

Not applicable.

## Supplementary Materials

The following supporting information can be downloaded at: https://www.mdpi.com/article/10.3390/diagnostics12040797/s1, Table S1: Cancer patient data; Table S2: Controls data.
